# CBM-I Training and Its Effect on Interpretations of Intent, Facial Expressions, Attention and Aggressive Behavior

**DOI:** 10.5964/ejop.2413

**Published:** 2021-05-31

**Authors:** Nouran AlMoghrabi, Ingmar H. A. Franken, Birgit Mayer, Menno van der Schoot, Jorg Huijding

**Affiliations:** Department of Psychology, Princess Nourah bint Abdulrahman University, Riyadh, Saudi Arabia; Department of Psychology, Education & Child Studies, Erasmus University Rotterdam, Rotterdam, the Netherlands; Department of Educational and Family Studies, Vrije Universiteit Amsterdam, Amersterdam, the Netherlands; Department of Child and Adolescent Studies, Utrecht University, Utrecht, the Netherlands; Edinburgh Napier University, Edinburgh, United Kingdom

**Keywords:** interpretation bias, attention bias, facial expressions, cognitive bias modification, aggression

## Abstract

There is abundant evidence suggesting that attention and interpretation biases are powerful precursors of aggression. However, little is known how these biases may interact with one another in the development and maintenance of aggression. Using cognitive bias modification of interpretation (CBM-I), the present study examined whether training more pro-social or hostile intent attributions would affect attention bias, interpretation bias of facial expressions, aggression and mood. University students (17–48 years) were assigned to either a positive training (n = 40), negative training (n = 40), or control training (n = 40). Results showed that the positive training successfully changed measures of intent attributions in a pro-social direction compared to the control training. The negative training changed measures of intent attributions in a hostile direction but not more so than the control training. We found no generalization of the training effects to relevant other outcomes. Possible explanations underlying these findings are discussed.

Theoretical models concerning the development of aggressive behavior, such as the Social Information Processing model (SIP; Crick & Dodge, 1994), suggest that aggressive individuals are characterized by biases in information processing, which contribute to the development and maintenance of aggression.

In support of the SIP model, research has found that aggressive individuals demonstrate biases in attention (for a review, see Wilkowski & Robinson, 2008) and interpretation (for a review, see de Castro, Veerman, Koops, Joop, & Monshouwer, 2002) compared to non-aggressive individuals. For instance, aggressive individuals show heightened attention for hostile cues compared to non-hostile cues. This bias in the allocation of attention toward hostile cues is believed to increase the likelihood to infer hostile intent (i.e., hostile attribution bias) even when the social situation is ambiguous, thus increasing the probability of an aggressive response. Additionally, deficiencies in encoding social cues and attributing hostile intent to others, does not only increase the likelihood of acting aggressively, but it also contributes to the maintenance of aggression. Thus, when others react aggressively in response to the aggressive act, it will play a role in confirming the aggressive individual’s initial hostile perception of the situation (Crick & Dodge, 1994).

These findings have led researchers to explore ways to modify such biases, using paradigms such as Cognitive Bias Modification (CBM), in order to examine the causal status of these biases in relation to aggression, and find targets for interventions (AlMoghrabi, Huijding, & Franken, 2018; AlMoghrabi, Huijding, Mayer, & Franken, 2019; Hawkins & Cougle, 2013; Vassilopoulos, Brouzos, & Andreou, 2015). To date, the majority of CBM studies on aggression have focused on modifying interpretation biases (CBM-I; AlMoghrabi et al., 2018; Hawkins & Cougle, 2013; Vassilopoulos et al., 2015).

In aggression studies, CBM-I consists of repeated practice on a specific task that is designed to modify interpretation of others’ intentions in social situations. The task typically comprises of emotionally ambiguous material to train participants to make more pro-social or hostile intent attributions using feedback (e.g., Vassilopoulos et al., 2015). Most CBM-I studies in aggression thus far used verbal stimuli such as vignettes. Typically, the vignettes describe hypothetical social situations in which one person “harms” another, while the intention of the harm-doer (i.e., provocateur) is ambiguous. Participants respond to questions about these situations that require them to make assumptions regarding the intention of the harm-doer, on which they subsequently receive feedback. By systematically reinforcing either pro-social or hostile intent attributions, participants’ interpretations are “trained” (or biased) in a certain direction. Participants subsequently complete self-report and/or behavioral aggression task, to examine the effect of the training. For example, one study found that compared to non-trained controls, a three-session attribution training program using flashcards was effective in increasing pro-social interpretation bias and decreasing hostile attributions regarding ambiguous social situations, anger and aggression in a sample of children high in aggression (Vassilopoulos et al., 2015).

Although such CBM-I studies have shown that aggression related interpretations can be successfully manipulated and affect aggression, a number of questions regarding CBM-I remain. First of all, the number of CBM-I studies in the context of aggression is limited. This calls up the question how robust the reported effects are and whether they can be replicated. Being able to draw more firm conclusions regarding the effects of the training procedure on aggression is important before applying such procedures to clinical samples. Second, although the SIP model postulates that cognitive biases in attention and interpretation are associated rather than independent, only one previous study examined the extent to which cognitive biases may be related, and jointly contribute to the maintenance of aggression (AlMoghrabi et al., 2019). It is possible that positive effects caused by CBM-I are not only specific to interpretation biases but can also affect other stages of the information processing, like encoding (i.e., attention). Interestingly, several anxiety studies provided evidence suggesting that change in one cognitive bias has significant effect on another cognitive bias (e.g., Amir, Bomyea, & Beard, 2010). AlMoghrabi and colleagues (2019) found that a CBM training of attention (CBM-A) that successfully increased attention allocation to cues that would help disambiguate the situation (i.e., adaptive cues) did not affect interpretation bias. To date, no aggression studies have focused on the question whether modification of interpretation bias can influence attentional deployment. This knowledge is relevant as this allows us to better understand to what extent these biases interact in the context of aggression, and might have practical implications for aggression reduction interventions targeting multiple cognitive biases.

A third question that has received little attention is what aspects of a social situation need to be targeted in CBM-I in order to successfully train intent attributions. Most studies to date used vignettes describing ambiguous social situations. Even though these vignettes lack much of the contextual information that is available in real life, this approach was used successfully to train more benign interpretations, and reduce anger and aggression (e.g., Vassilopoulos et al., 2015).

Another approach has been to directly target the interpretation of facial expressions, based on the idea that these are particularly salient cues for making inferences about other people’s intentions. As expected, participants that were trained by reinforcing the perception of happiness over anger in ambiguous facial expressions, reported less anger and showed less aggressive behavior (Penton-Voak et al., 2013). Interestingly however, a recent study found that a successful manipulation of the interpretation of facial expressions did not affect interpretations of intent in a different task (Hiemstra, de Castro, & Thomaes, 2018).

A final approach that has been used is to modify hostile attribution biases using visual stimuli instead of vignettes (AlMoghrabi et al., 2018), based on the idea that compared to verbal cues, visual cues (e.g., facial and physical expressions) are more meaningful in social interactions as they carry important information regarding the intentions of others (Cadesky, Mota, & Schachar, 2000). The results indicated that the positive training with visual stimuli led to an increase in pro-social interpretation bias and decrease in anger and verbal aggression. However, it remained unclear whether the training also impacted on participants’ interpretations of facial expressions. This is an important question, as it gives insight into what factors may contribute to the training effects and might provide cues on how the effects of training might be strengthened.

To address these outstanding questions we aimed to examine whether: 1) we could replicate our earlier findings that positive training with visual stimuli led to an increase in pro-social interpretation bias and decrease in anger and verbal aggression (AlMoghrabi et al., 2018); 2) any CBM-I induced changes in interpretation biases affect attention allocation; and 3) any changes in interpretation biases of intent affect interpretation bias of facial expressions.

In this study, participants completed one of three training conditions. In the positive training, participants were trained to interpret the intention of the harm-doer as pro-social. In the negative training, participants were trained to interpret the intention of the harm-doer as hostile. In the control condition, participants completed a neutral training.

We expected that: 1) the positive training would increase pro-social intent attribution bias from pre-to post treatment relative to a neutral training, and decrease self-reported aggression. While the negative training would increase hostile attribution bias from pre-to post treatment relative to a neutral training, and increase self-reported aggression; 2) the positive training would lead to heightened attention to the facial expression of the harm-doer (i.e., adaptive cue that would help disambiguate the situation in a pro-social way), while the negative training would lead to heightened attention to the negative outcome of the situation (i.e., maladaptive cue that would help disambiguate the situation in a hostile way), and 3) the positive training would increase pro-social interpretation bias of facial expressions from pre-to post treatment relative to the neutral training, whereas the negative training would increase hostile interpretation bias of facial expressions relative to the neutral training.

## Method

### Participants

Sixty male and 60 female students from Erasmus University Rotterdam (79 Caucasians, seven Asian, six Middle Eastern, six Hispanic, three African, and 19 others), aged between 17 and 48 (*M* = 21.44, *SD* = 4.27) participated in exchange for course credits. The study was conducted in compliance with the Declaration of Helsinki (World Medical Association, 2001).

### Stimulus Materials

**Figure 1:**
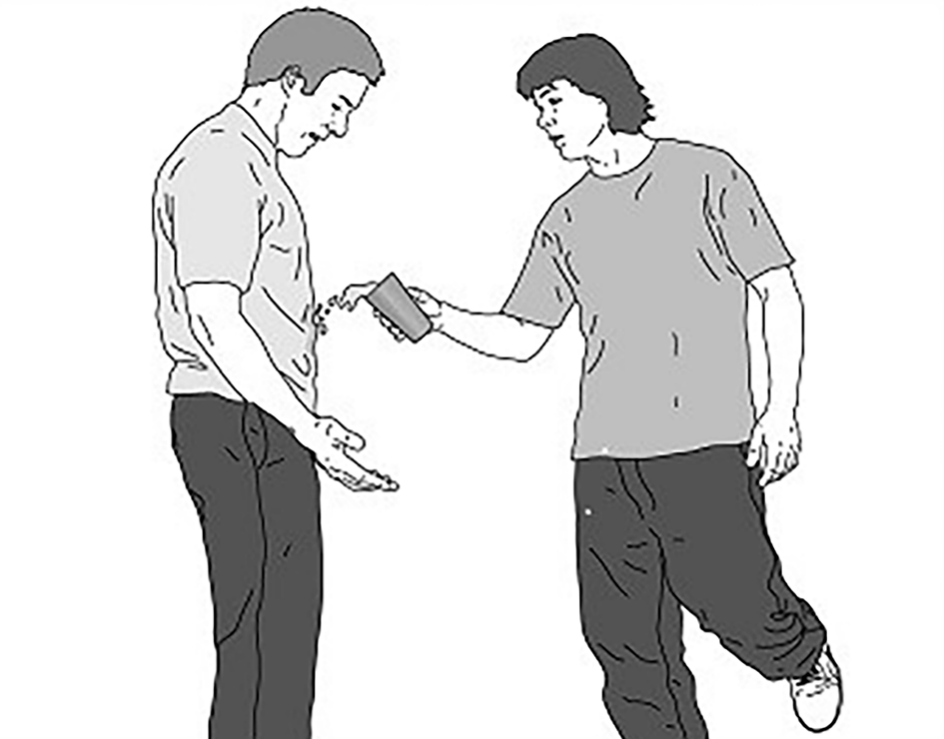
Example Image From the Baseline Phase

**Figure 2:**
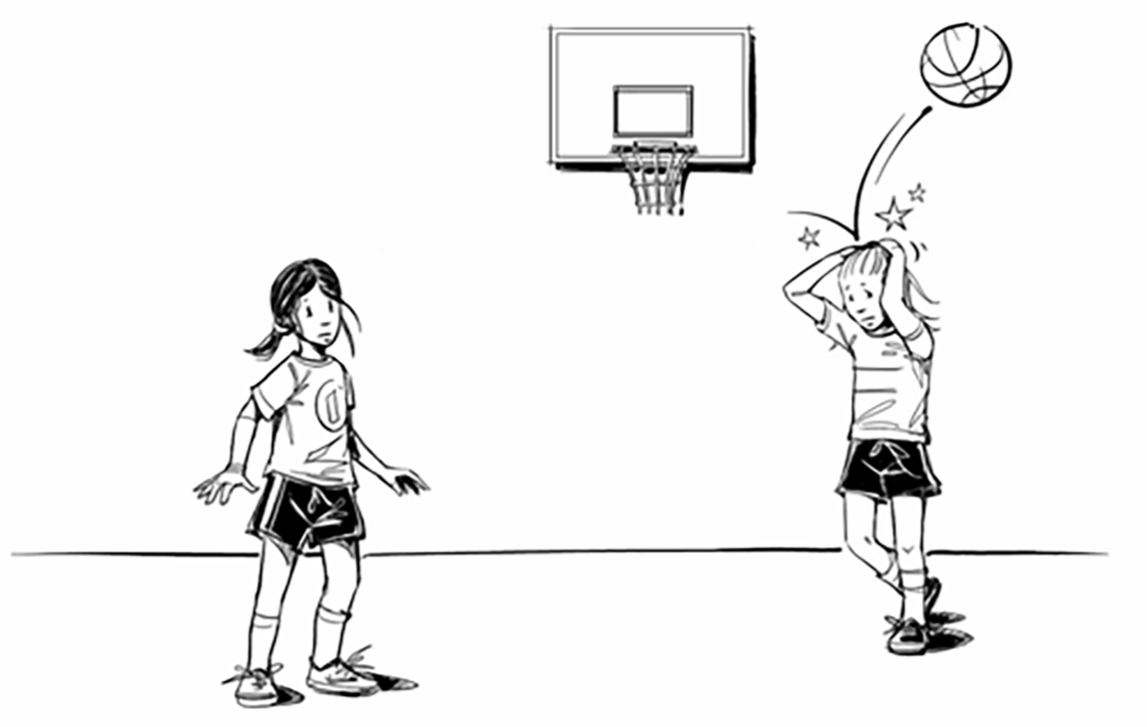
Example Image From the Training Phase

For the baseline and test phases, a set of 12 pictures was used that were originally from the study of Wilkowski, Robinson, Gordon, and Troop-Gordon (2007; see Figure 1) and were previously used in a similar study by AlMoghrabi et al. (2018). For the training phase, we selected images that were originally developed by Horsley, de Castro, and Van der Schoot (2010; see Figure 2), supplemented by 19 pictures that were previously used in the study of AlMoghrabi et al. (2018) and additional 21 pictures collected via stock image websites. All pictures described interactions between two characters. One of the two characters (i.e., harm-doer) initiates a hostile behavior that negatively affects the other character (i.e., victim). Most importantly, the facial expression of the character that initiated the harm should be ambiguous or neutral to not give a definite clue that the act of the harm-doer is either intentional or unintentional.

The stimulus materials in the training phase were pilot tested in a previous study (AlMoghrabi et al., 2018). In this study, students (*n* = 40) were asked to rate the extent to which the depicted harm was intentional and as well as aggressiveness of the facial expression of the harm-doer. Participants rated intentionality on a 100-point Visual Analogue Scale (VAS) that was anchored with the labels “Accidental” on the left and “Intentional” on the right end. Additionally, participants rated the facial expressions of the harm-doer on a 100-point VAS that was anchored with the labels “Friendly” on the left and “Aggressive” on the right end. The results of the pilot were as expected, the pictures in the training phase were rated on average as ambiguous for the intent of the harm-doer (*M* = 50.3, *SD* = 8.5), and ambiguous, but leaning a bit towards friendly, for the facial expression of the harm-doer (*M* = 42.6, *SD* = 5.1).

### CBM-I Training

The CBM-I training was presented using E-prime software (Version 2.0; Psychology Software Tools, 2002) for Windows and consisted of four phases: practice, baseline, training, and test. The practice phase was implemented to introduce participants to the experimental procedure and consisted of three trials. In order to examine the effects of the training on interpretation and attention bias, an assessment of interpretation and attention bias was administered during the baseline and test phases. The baseline and test phases were identical and consisted of six trials each. The manipulation of interpretation bias took place during the training phase, which consisted of 40 trials. The whole CBM-I task took approximately 25 minutes to complete.

#### Phase 1 (Practice)

On each trial participants were presented with an image which was not related to the images used in the assessment or training phases. Participants were instructed to fix their gaze on a certain area of interest (AOI) and received feedback on their performance.

#### Phase 2 (Baseline) and 4 (Test)

On each trial participants were presented with an image of a social situation (see Figure 1 for an example). In total there were 12 images for the assessment phases, half of the participants within each condition received images 1–6 at baseline and images 7–12 at test phase, whereas this order was reversed for the other half of the participants.

To measure attention bias, participants were asked to look at the picture closely. For each image, AOIs were defined around a “maladaptive” cue showing the negative outcome of the situation (e.g., water spilling on the victim’s clothes), and an “adaptive” cue (the face of the harm-doer, see Figure 1). While looking at the images, participant’s eye movements were recorded automatically using an eye tracking device. As a measure of the attention bias, we used the total dwell time on both AOI (i.e., adaptive and maladaptive cues) that were presented during a trial, which reflects the total time a participant spent looking at a certain AOI.

Thereafter, in order to measure interpretation bias regarding the intention of the harm-doer (IB-intent), participants were asked to answer the question “Did this happen by accident or intentionally?” Participants indicated the likelihood that the incident happened by accident or intentionally by dragging an arrow on a 100-point VAS that was anchored with the labels “*Very likely accidental*” (−50) on the left and “*Very likely intentional*” (+50) on the right end of the scale. To assess interpretation of facial expressions of the harm-doer, participants answered the question “How friendly or aggressive is the facial expression of the harm-doer?” by dragging an arrow on a 100-point VAS that was anchored with the labels “*Very friendly*” (−50) on the left and “*Very Aggressive*” (+50) on the right end of the scale. Finally, participants rated the question (i.e., perceived anger), “How would you feel if this happened to you” by dragging an arrow on a 100-point VAS that was anchored with the labels “*Very happy*” (−50) on the left and “*Very angry*” (+50) on the right end of the scale. During this phase, no feedback was provided. The viewing time was fixed for 5,000 ms for each image.

#### Phase 3 (Training Phase)

On each trial participants were presented with an image of a social situation. The images were always preceded with a short description of the situation. For example, the image presented in Figure 2, was accompanied by the description “The ball hits her head hard.” Following this sentence, another (filler) sentence appeared on the screen that, depending on the condition, disambiguated the situation in a hostile or non-hostile manner. To ensure that participants read this information, one letter was missing from the key word of this filler sentence, and participants were required to fill in the missing letter (adapted from Hawkins & Cougle, 2013). For instance, in the positive condition one disambiguating sentence read “It was accident_l,” while in the negative condition it read “She attac_ed her.” The image and both sentences (description and filler sentence) were presented together on the screen below the image. After the participant pressed the correct letter on the keyboard to complete the filler sentence, both sentences remained on the screen for another 2,000 ms and then disappeared. Next, this interpretation was reinforced by asking participants to answer “Yes” or “No” to a comprehension question “Was she determined to hit her hard with the ball?” To reduce the expectancy effects across all conditions, the comprehension questions were phrased in such a way that for half of them the correct answer would be “Yes” and for the other half “No.” If the participant answered the comprehension question correctly the word “CORRECT” was presented at the top of the screen in bold green font. If the participant’s answer was incorrect the word “INCORRECT” was presented at the top of the screen in bold red font. The feedback remained on the screen for 1,500 ms, after which the next trial began.

Participants in the control group were presented with the same pictures, read the same descriptive sentences, and were also asked to fill in the missing letter in the sentence and answer the comprehension question. However, the sentences with the missing letter and the comprehension questions were unrelated to the intent of the harm-doer or the incident in the picture.

### Eye Tracking Assessment

Eye movements were assessed at practice, baseline and test phase using SMI-RED 250 device (Sensomotoric Instruments GmbH, Teltow, Germany) with a sampling rate of 250 Hz.

The stimuli were presented on a 22-inch computer screen with the resolution set to 1,680 × 1,050 pixels. The viewing distance was approximately 60 cm. The size of the image was 1,344 × 777 pixels. Each AOI was defined as a square area and had a size of either 252 × 210 or 336 × 210 pixels to encompass the entire area of display of the adaptive or maladaptive cue in the picture. To ensure accuracy of the gaze pattern, a nine-point calibration and 4-point validation was performed before starting with the first phase.

### Questionnaires

We assessed state aggression using a reworded version of Buss and Perry’s (1992) trait Aggression Questionnaire (AQ; c.f. Farrar & Krcmar, 2006). Pre-training, the modified questionnaire started with the following instruction: “Imagine that you just bought something to drink. When you walk outside, somebody bumps into you, spilling your drink over your favorite clothes. As you look at the mess, you hear this person swearing” Post-training the instruction was: “Imagine that you are at Starbucks working on an assignment. Suddenly, someone bumps into your table, spilling coffee all over your notes. You see that the other person looks really annoyed” Additionally, the items comprised of items from the AQ that were rephrased. For example, the original AQ item “If someone hits me, I hit back” was rephrased to “If this person hits me, I'd hit back” to match state aggression. For each of the items, the participants were instructed to indicate their response on the items form 1 = *extremely uncharacteristic of me* to 7 = *extremely characteristic of me*. In total, 20-items were modified from the original AQ on three subscales: physical aggression, verbal aggression, and anger. Total score was used, with higher scores meaning higher state aggression. In the current sample, Cronbach’s alpha was .88 pre-training and .90 post-training.

The Reactive-Proactive Aggression Questionnaire (RPQ; Raine et al., 2006) was used to assess both reactive (11-items; e.g., “damaged things because you felt mad”) and proactive (12-items; e.g., “taken things from other students”) aggression. Participants provided a rating of 0 = *never*, 1 = *sometimes*, and 2 = *often* for each item. In the current sample, Cronbach’s alpha was .67 for reactive and .51 for proactive aggression.

Anger was measured using part B of the Novaco Anger Scale (NAS; Novaco, 1994). This questionnaire consists of 25 potentially-provoking situations that are rated on a 5-point scale from 0 = *little annoyance* to 4 = *very angry*. In the current sample, Cronbach’s alpha was .90. Additionally, the Hostility Aggression trait subscale (8-items) from the AQ (Buss & Perry, 1992) was added post-training. In the current sample, Cronbach’s alpha was .81. To assess hostile interpretation bias post-training, the Word Sentence Association Paradigm-Hostility (WSAP-H; Dillon, Allan, Cougle, & Fincham, 2016) was used. The WSAP-H is a computerized measure that presents participants with 16-ambiguous interpersonal scenarios (e.g., “Someone frowns at you”) on a computer screen together with a word. Participants are instructed to rate how well the word is related to the scenario by dragging an arrow on a 100-point VAS that was labeled “Not at all related” and “Very related” at the extreme ends of the scale. Each scenario was presented twice in a random order, once paired with a benign-related word (e.g., “Unhappy”) and once paired with a hostile-related word (e.g., “Hostile”). Average ratings were calculated separately for both the hostile and benign words; higher scores on these two measures indicated a higher hostile or a higher pro-social interpretation bias, respectively. In the current sample, Cronbach’s alpha was .84 for the hostile subscale and .87 for the benign subscale.

Mood was assessed pre and post training, using a 100-point VAS labeled 0 = *not at all* and 100 = *very much* at the extremes. Participants rated how they felt at the moment (afraid, happy, sad, and angry) by dragging on each mood scale, an arrow to the point on the scale that best reflected their current mood. In addition, the Positive Affect and Negative Affect Schedule (PANAS; Watson, Clark, & Tellegen, 1988) was administered post-training to assess the presence of negative and positive affect. Participants had to rate how much they generally feel (1 = *slightly*, 5 = *extremely*) about 10-positive emotional states (e.g., inspired), and 10-negative states (e.g., upset). Additionally, five items (e.g., snappy) specifically covering anger were added to the original negative affect measure. In the current sample, Cronbach's alpha for positive affect was .85, and for negative affect .89.

### Procedure

The participants were randomly assigned to one of three conditions: the positive condition (*n* = 40), the negative condition (*n* = 40), or control condition (*n* = 40). For either condition, participants started by completing the AQ, RPQ, and mood questionnaires. Then they received specific instructions regarding the eye-tracking and the CBM-I training. Finally, the participants completed the AQ, WSAP-H, PANAS, NAS, and mood questionnaires. The entire experiment took approximately 60 minutes to complete.

### Data Reduction

For each condition, separate mean scores for the VAS likelihood ratings of the pre- and post-training interpretation bias (IB) scores for the pre- and post-training assessments regarding participants’ interpretation of intent and facial expression of the harm-doer were computed. Thus, higher IB-intent and IB-facial expression scores indicate that hostile interpretations were rated as more likely to be true than pro-social interpretations. In a similar way, for each condition, separate mean scores for the VAS likelihood ratings of the pre- and post-training perceived anger score were computed. Higher scores on perceived anger indicated that participants are more likely to feel angry than happy in the presented situation. For the self-reported interpretation bias measure, the WSAP-H, we first calculated a separate mean score for both the hostile and benign interpretations. Next, to create a WSAP-H IB score, we subtracted the mean score of the hostility subscale from the mean score of the benign subscale. Thus, positive WSAP-H (IB) scores indicate that positive interpretations were rated as more likely to be true than the negative interpretations.

Attention bias scores were calculated using gaze data collected by the eye-tracker. A minimum amount of eye gaze time of 80 ms at a certain AOI was qualified as a gaze fixation (e.g., Gerdes, Alpers, & Pauli, 2008).

Next, we calculated separate mean total viewing times in ms for the pre-defined AOI for the adaptive and the maladaptive cues at pre- and post-training. Next, pre- and post-training attention bias (AB) scores were calculated by subtracting the mean total viewing time at the maladaptive cues from the mean total viewing time at the adaptive cues. Thus, higher AB scores indicate more attention allocation to adaptive (facial) than to maladaptive (negative outcome) cues.

## Results

### Preliminary Analysis

In order to ascertain the appropriateness of our IB measure, we correlated the IB-intent scores for the pre- and post-training assessments with the concurrently assessed aggression and hostility-related measures (i.e., AQ, NAS, RPQ, WASP-H, and VAS anger). Only IB-intent post-training scores correlated significantly with WSAP-H (IB) (*r* = −.35, *p* < .001).

In addition, IB-facial expression pre-training scores correlated significantly with the AQ (*r* = .20, *p* = .028) specifically with the physical subscale (pre: *r* = .30, *p* = .001, post: *r* = .178, *p* = .051). IB-facial expression post-training scores correlated significantly with AQ score post-training (*r* = .183, *p* = .046), specifically physical (*r* = .201, *p* = .027). Additionally, the pre- and post-IB-facial expressions scores correlated significantly with WSAP-H (IB) (pre: *r* = −.28, *p* = .002, post: *r* = −.24, *p* = .008) and NAS (pre: *r* = .19, *p* = .043, post: *r* = .24, *p* = .008). This provides some support for the validity of our approach as it shows that we assessed and trained interpretations that are meaningfully related to aggression.

Finally, to get an idea of whether the training approach was clear and doable for participants, we explored participants’ accuracy during training. While participants in the positive (*M* = 14.12%, *SD* = 9.40) and control condition (*M* = 9%, *SD* = 4.19) made few errors this was not the case in the negative condition, in which significantly more errors were made (*M* = 27.12%, *SD* = 18.12), *F*(2, 117) = 22.89, *p* < .001, $\eta_{p}^{2}$ = .28. The high number of errors in the negative training seems to suggest that a number of participants in the current study resisted the negative training by insisting on choosing the prosocial interpretation despite receiving negative feedback.

### Baseline Measures

Descriptive statistics for the baseline measures are presented in Table 1. There were no significant differences between the participants in the positive, negative and control conditions in their baseline levels of self-reported aggressive behavior (AQ and RPQ) and mood ratings (afraid, happy, sad, and angry), for all *F*(2, 117) < 1.98, *p* > .272, $\eta_{p}^{2}$ < .013. Also, there were no significant differences between the training conditions in their pre-training IB-Intent, *F*(2, 117) = .111, *p* = .895, $\eta_{p}^{2}$ = .00. However, compared to participants in the negative condition, participants in the control condition reported a higher level of pre-training hostile IB-facial expressions, *F*(2, 117) = 3.16, *p* = .038, $\eta_{p}^{2}$ = .05. In addition, they showed a tendency to higher attention bias to adaptive cues (i.e., the face of the harm-doer) pre-training, *F*(2, 117) = 3.18, *p* = .059, $\eta_{p}^{2}$ = .05, and scored higher on the pre-training AQ anger subscale compared to participants in the negative condition, *F*(2, 117) = 3.34, *p* = .035, $\eta_{p}^{2}$ = .05.

**Table 1:** Descriptive Statistics for Baseline Measures

Measure	Positive training		Negative training		Controltraining	
	*M*	*SD*	*M*	*SD*	*M*	*SD*
**Pre-training**						
Aggression Questionnaire	58.13	14.73	55.68	19.78	62.90	14.46
Physical Aggression	21.13	8.38	22.18	9.42	24.07	7.99
Verbal Aggression	16.10	4.65	15.00	6.14	16.90	4.88
Anger	20.90	5.32	18.50	7.01	21.93	5.80
Reactive Aggression	18.20	2.95	18.40	2.69	13.85	1.72
Proactive Aggression	13.53	1.43	13.38	1.61	18.55	2.86
VAS Anger	8.65	17.75	8.20	15.82	9.18	15.08
VAS Fear	4.85	10.32	7.45	14.00	8.00	11.32
VAS Sadness	11.75	15.53	16.35	20.05	14.05	17.46
VAS Happiness	65.73	20.95	62.82	19.23	61.35	19.53

*Note.* VAS = Visual Analogue Scale.

### Effects of Training on Interpretation Bias

**Figure 3:**
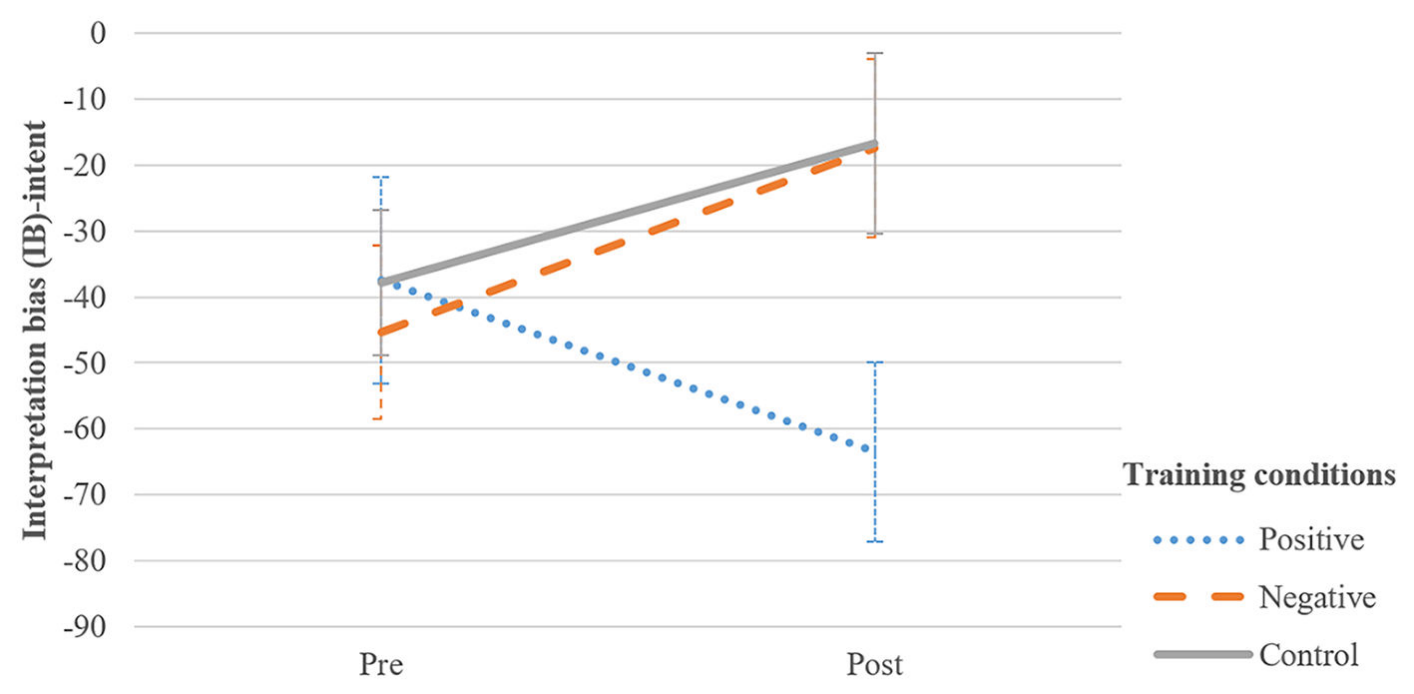
Average Sum Scores of IB − Intent at Pre− and Post−Training for Each Training Condition *Note.* Error bars indicate standard error of the mean. Higher IB-intent scores indicate that hostile interpretations of intent were rated as more likely to be true than pro-social interpretations of intent.

A 2 Assessment (pre- vs. post-treatment) × 3 Group (negative, positive and control training) mixed analysis of variance (ANOVA). The analysis showed no significant main effects for both Group, *F*(2, 117) = 1.35, *p* = .265, $\eta_{p}^{2}$ = .02, and Assessment, *F*(1, 117) = .64, *p* = .425, $\eta_{p}^{2}$ = .01. However, the crucial interaction between Group and Assessment was significant, *F*(2, 117) = 3.15, *p* = .047, $\eta_{p}^{2}$ = .05 (see Figure 3). Paired-samples *t*-tests of change over time showed that change in hostile IB-intent from pre- to post-training was not significant for the positive *t*(39) = 1.68, *p* = .101 and control condition *t*(39) = −1.12, *p* = .268. For the negative condition, the increase in hostile IB-intent from pre- to post-training approached significance, *t*(39) = −1.84, *p* = .074.

Follow-up analysis revealed that the groups differed significantly in IB-intent post-training. The positive condition differed significantly from the negative condition, *F*(2, 117) = 3.87, *p* = .024, $\eta_{p}^{2}$ = .06. Also, the positive condition differed significantly from the control condition, *F*(2, 117) = 3.87, *p* = .044, $\eta_{p}^{2}$ = .06. However, the negative and control condition were not significantly different, *F*(2, 117) = 3.87, *p* = .999, $\eta_{p}^{2}$ = .06.

**Figure 4:**
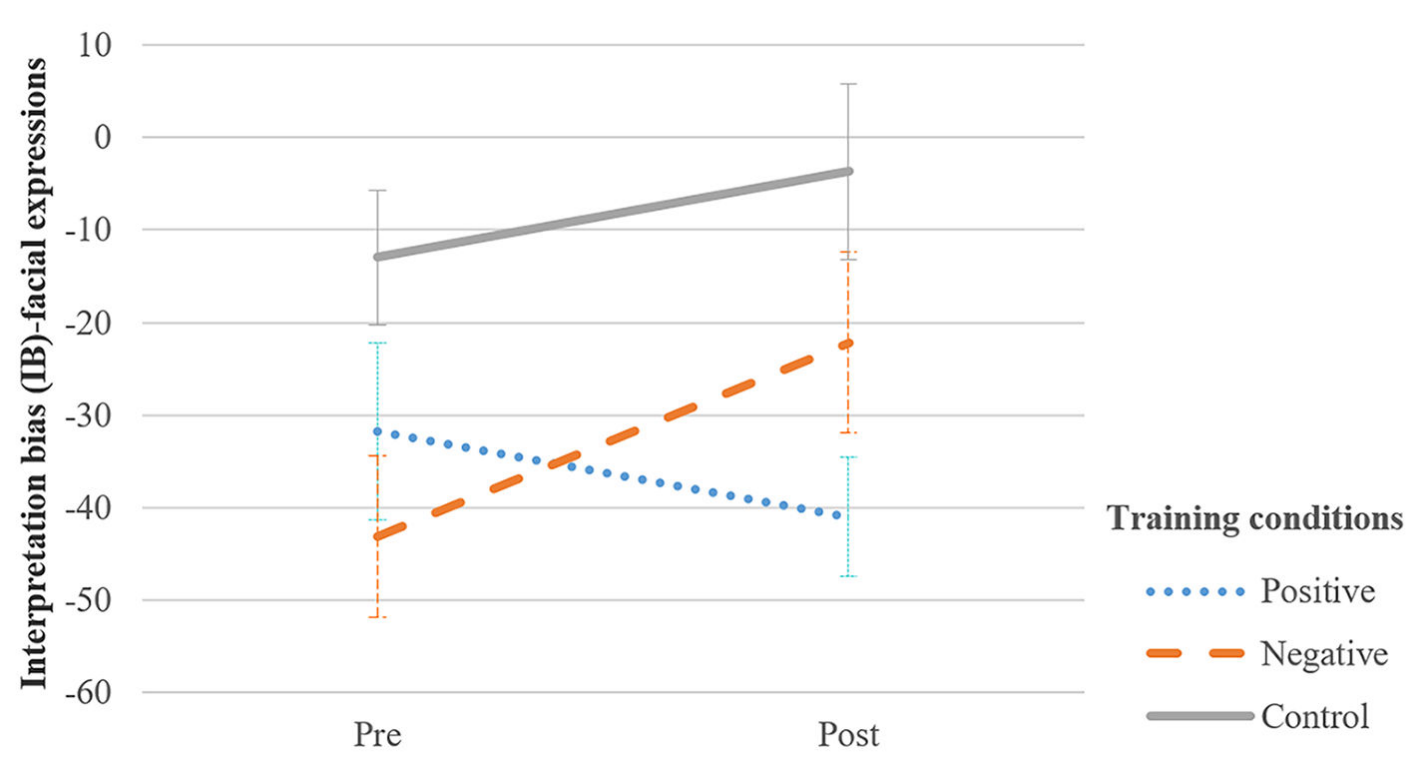
Average Sum Scores of IB − Facial Expressions at Pre− and Post−Training for Each Training Condition *Note.* Error bars indicate standard error of the mean. Higher IB-facial expressions scores indicate that hostile interpretations of facial expressions were rated as more likely to be true than pro-social interpretations of facial expressions.

To examine the effects of the interpretation bias training on IB-facial expressions, the IB (facial expressions) scores were subjected to a 2 Assessment (pre- vs. post-treatment) × 3 Group (negative, positive and control training) mixed ANOVA. The analysis revealed that the crucial interaction between Group and Assessment was not significant, *F*(2, 117) = 2.20, *p* = .115, $\eta_{p}^{2}$ = .04 (see Figure 4). Moreover, no significant main effect for Assessment emerged, *F*(1, 117) = 1.41, *p* = .238, $\eta_{p}^{2}$ = .01. There was, however, a significant main effect of Group, *F*(2, 117) = 4.82, *p* = .010, $\eta_{p}^{2}$ = .08.

To examine the training effects on perceived anger in response to potentially provocative social situations, the perceived anger scores were subjected to a 2 Assessment (pre- vs. post-treatment) × 3 Group (negative, positive and control training) mixed ANOVA. The analysis revealed that the crucial interaction between Group and Assessment was not significant, *F*(2, 117) = 1.53, *p* = .220, $\eta_{p}^{2}$ = .03. Moreover, no significant effects for Group emerged, *F*(2, 117) = .06, *p* = .943, $\eta_{p}^{2}$ = .00. However, the main effect of Assessment was significant, *F*(1, 117) = 15.35, *p* < .001, $\eta_{p}^{2}$ = .12. Overall, Anger scores decreased from pre- to post-training.

Additionally, we examined the effect of the CBM-I training on the WSAP-H (IB) as a measure of hostile interpretation bias. There was no significant difference between the three training conditions, *F*(2, 117) = 1.10, *p* = .335, $\eta_{p}^{2}$ = .02.

### Effects of Training on Attention Bias

**Figure 5:**
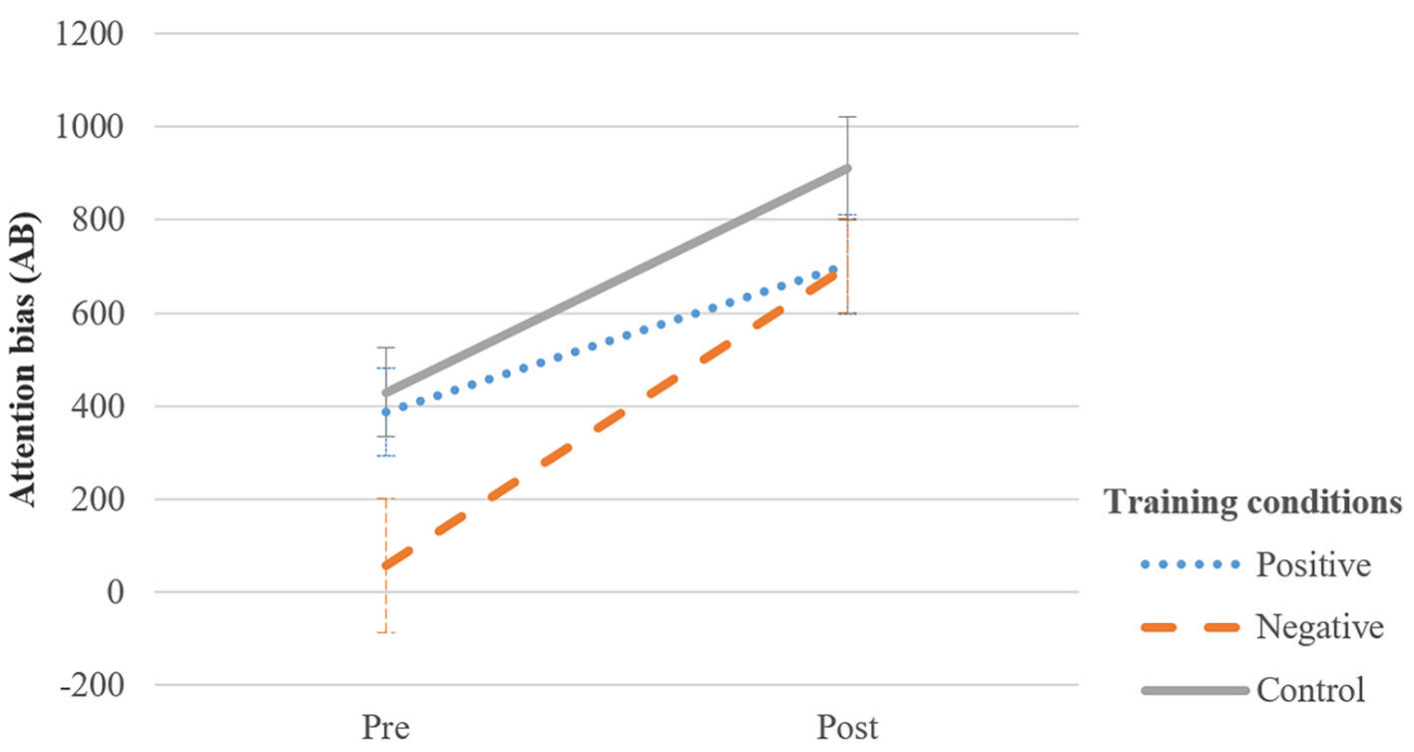
Average Attentional Bias Scores at Pre− and Post−Training for Each Training Condition *Note.* Error bars indicate standard error of the mean. Higher AB scores indicate that more attention allocation to adaptive (facial) than to maladaptive (negative outcome) cues.

A 2 Assessment (pre- vs. post-treatment) × 3 Group (positive, negative and control training) mixed ANOVA. The analysis revealed that the crucial interaction between Group and Assessment was not significant, *F*(2, 117) = 1.53, *p* = .221, $\eta_{p}^{2}$ = .03. Moreover, no significant effects for Group emerged, *F*(2, 117) = 2.72, *p* = .070, $\eta_{p}^{2}$ = .04. However, the main effect of Assessment was significant, *F*(1, 117) = 39.66, *p* < .001, $\eta_{p}^{2}$ = .25 (see Figure 5). Surprisingly, it was found that in all training conditions attention bias became significantly more positive from pre- to post-training, indicated by relatively longer fixation durations on the adaptive cues after training.

### Effects of Training on Mood

VAS state mood ratings (afraid, happy, sad, and angry) were subjected to separate 2 Assessment (pre- vs. post-treatment) × 3 Group (positive, negative and control training) mixed ANOVAs. Only a significant main effect of Assessment emerged for self-reported sadness, *F*(1, 117) = 4.19, *p* = .043, $\eta_{p}^{2}$ = .04 and self-reported fear, *F*(1, 117) = 10.07, *p* = .002, $\eta_{p}^{2}$ = .08, indicating that for all training conditions the VAS scores decreased from pre- to post-training. Neither the crucial interaction, for all *F*(2, 117) < .75, *p* > .477, $\eta_{p}^{2}$ < .01, nor the main effect of Group, for all *F*(2, 117) < .63, *p* > .534, $\eta_{p}^{2}$ < .01, was found significant.

In addition, the training conditions did not differ on the PANAS scores. The ANOVA results confirmed that the positive, negative and control condition didn’t differ significantly in terms of either their positive, *F*(2, 117) = .15, *p* = .860, $\eta_{p}^{2}$ = .00 or negative trait affect scores, *F*(2, 117) = .81, *p* = .449, $\eta_{p}^{2}$ = .01.

### Effects of Training on Aggression

Participants scores from the AQ were subjected to a 2 Assessment (pre- vs. post-treatment) × 3 Group (positive, negative and control training) mixed ANOVA. The analysis revealed no significant interaction between Group and Assessment for the AQ and its subscales, for all *F*(1, 117) < 2.44, *p* > .091, $\eta_{p}^{2}$ > .02. Only a significant main effect of Assessment emerged for AQ total scores, *F*(1, 117) = 12.16, *p* = .001, $\eta_{p}^{2}$ = .09, indicating that over-all self-reported aggression significantly increased from pre- to post-training. Moreover, for the AQ subscales the results showed a significant effect for main effects of Assessment for anger, *F*(1, 117) = 6.42, *p* = .013, $\eta_{p}^{2}$ = .05, and a trend towards significance for Group, *F*(2, 117) = 2.91, *p* = .058, $\eta_{p}^{2}$ = .05., with higher scores from pre- to post-training and higher overall scores for the negative and control condition. Also, main effects of Assessment emerged for physical aggression, *F*(1, 117) = 12.02, *p* = .001, $\eta_{p}^{2}$ = .09, indicating that overall self-reported anger and physical aggression significantly increased from pre- to post-training. Next, participants’ scores on the post-training AQ hostility subscale were compared between the three conditions. The ANOVA results showed that the groups differed on the hostility subscale, *F*(2, 117) = 6.22, *p* = .003, $\eta_{p}^{2}$ = .10. Specifically, there was a marginally significant difference between the control and positive condition, *F*(2, 117) = 6.22, *p* = .050, $\eta_{p}^{2}$ = .10, and significant difference between the control and negative condition, *F*(2, 117) = 6.22, *p* = .002, $\eta_{p}^{2}$ = .10, respectively. This indicates that the participants in the control condition scored higher on the hostility subscale compared to the participants in the positive and negative condition. However, there was no significant difference between the positive and negative condition on the hostility subscale, *F*(2, 117) = 6.22, *p* = .534, $\eta_{p}^{2}$ = .10.

We performed an additional analysis to test the relation between AQ and its subscales with IB-intent and IB-facial expression. First, we calculated IB-intent and IB-facial expression change scores by subtracting the bias score before the training from the bias score after the training. Thus, more positive bias change scores indicate that participant’s interpretations of intent and facial expression became more hostile. Second, we calculated a change score for the AQ total score and its subscales by subtracting the aggression score before the training from the aggression score after the training. Thus, more positive scores indicate that participants become more aggressive. Next, we correlated the change in bias and perception scores with the change scores of the AQ and its subscales.

Only the change score for IB-facial expression showed a significant positive correlation with the AQ total score with the negative condition (*r* = .39, *p* = .013). In addition, the change score for IB-facial expression was not significantly related to AQ total score within the positive (*r* = .12, *p* = .446) and control condition (*r* = −.00, *p* = .980). The same was true for the correlations with the AQ subscales except for physical aggression, which showed a significant positive correlation with IB-facial expression within the positive (*r* = .32, *p* = .042) and negative (*r* = .37, *p* = .018), but not within the control condition (*r* = .06, *p* = .703). This suggests that the more the facial expressions of the harm-doer were interpreted in a hostile way, the more physical aggression participants reported after the training (see Table 2).

**Table 2:** Correlations Between Change Scores of Interpretation Bias of Intent and Facial Expressions With Change Scores of the Aggression Questionnaire and Its Subscales

Measure	Interpretation bias of intent/facial expressions		
	Positive training	Negative training	Control training
Aggression Questionnaire	.09/.12	.05/.39*	.02/−.00
Physical Aggression	.16/.32*	.12/.37*	.03/.06
Verbal Aggression	−.05/−.13	−.06/.26	−.09/−.14
Anger	.05/.01	.05/.28	.06/−.00

*p < .05.

Finally, participant’s anger scores on the NAS were compared between the three conditions. The results showed that the groups did not differ in terms of their NAS scoring *F*(2, 117) = 0.74, *p* = .480, $\eta_{p}^{2}$ = .01.

## Discussion

The current study examined three questions. First, we investigated whether a cognitive bias modification of interpretation (CBM-I) procedure using pictorial stimuli- change interpretations and whether those changes in turn influences attention bias. Second, we looked at whether any changes in interpretation bias of intent influences interpretation bias of facial expressions. Finally, we examined whether we could replicate our earlier findings that the current CBM-I procedure would lead to a reduction in self-reported anger and aggression, and an increase on positive mood.

To start with the first question, the results indicated that our positive CBM-I training successfully changed measures of interpretation bias of intent in a pro-social direction compared to a control training. The negative CBM-I training, changed measures of interpretation bias of intent in a hostile direction but not more so than a control training. These results are well in line with previous findings demonstrating that aggression-related interpretation biases can be trained (AlMoghrabi et al., 2018; Hawkins & Cougle, 2013; Vassilopoulos et al., 2015). However, the change in interpretation bias of intent did not seem to have influence on attention bias as the results from the eye-tracking data indicate that in all training conditions attention bias became more positive. Although there is some evidence suggesting interrelations between interpretation and attention biases and that training one bias can affect the other bias (Amir et al., 2010; White et al., 2011), we were not able to replicate this finding in the present study. There are a number of possible explanations for this. First, although the crucial interaction between training condition and assessment (i.e., pre- and post-training) was significant, and that the change of interpretation bias of intent went in the intended direction, post-hoc analysis showed that participants’ interpretation bias of intent scores did not change much from pre- to post-training. Also, the analysis showed that participants’ interpretation bias of intent in the negative training condition was not significantly different from the control condition. Thus, it is possible that the modest effect of the CBM-I on interpretation bias of intent limited the transfer effect of the modified interpretations to attentional processes. A possible explanation for the modest effect of the CBM-I training on interpretation bias of intent may lie in the number of sessions and trials of the training. Although experimental CBM training studies varies in the number of trials and sessions, it has been suggested that multiple-session training produce greater bias change (Hallion & Ruscio, 2011). In the current study participants completed a total number of 40 training trials during a single session. Thus, it might be necessary with CBM training paradigms with limited number of trials, to increase the number of training sessions to yield stronger training effect on cognitive biases. Second, the lack of interaction between attention and interpretation biases in the current study and in the previous study of ours (AlMoghrabi et al., 2019), points towards the possibility that one cognitive bias does not influence the other and that each of these cognitive biases function as a distinct process in the context of aggression. This latter explanation is well in line with a study by Maoz et al. (2017) who examined the relation between attention and interpretation biases in the context of anger by using emotional facial expressions. The results indicated that attention and interpretation biases did not show an interaction effect suggesting that it is possible that both these biases function in isolation of one another in its contribution to anger. However, the discrepancy in findings suggests further work is needed. It may be the case that in aggressive participants (not represented in the current study) a stronger modification effect on interpretation bias may occur, and in turn these two biases might interact and affect each other.

Regarding the second question, the results showed that the change in interpretation bias of intent did not lead to a change on rating ambiguous facial expressions from pre- to post-training for any of the training conditions. This might be due to the fact that changes in interpretation bias of intent in the current study were not as strong as we would have predicted. However, both interpretation bias of intent and interpretation bias of facial expressions were in the same direction for all training conditions, which could suggest that both these biases are closely related. Our finding is in contrast with the study of Hiemstra et al. (2018), where they successfully modified hostile interpretations of facial expressions in an aggressive sample. However, those changes did not generalize to changes on participants’ interpretation of intent in a game context. These contrasting results might be explained by differences in the stimulus material in both studies. While Hiemstra et al. (2018) used morphed faces to modify interpretation bias of facial expressions, our stimulus materials did not only include the face of the characters but also included a full scene of the characters interacting with each other with clear body expressions. This might influence how participants interpret the facial expressions of those characters. At this stage this is only speculation, and the association between interpretation of intent and interpretation of facial expressions should still be tested in future work. It is possible that in real-life social interactions aggressive behavior may emerge not only through hostile interpretation of intent. It could also occur simultaneously with misinterpreting facial expressions, and that both these misinterpretations function as a driving force for aggressive responses. Thus, understanding the role of facial expressions when interpreting others’ intentions may improve future CBM-I intervention programs.

Regarding the third question, based on SIP theories, that suggest that cognitive biases are causally related to aggressive behavior by influencing how ambiguous social information is processed (Crick & Dodge, 1994), we expected that changes in interpretation bias of intent would affect aggression. In contrast, the CBM-I training did not have any effect on mood, anger and aggression measures post-training. Additionally, it did not result in the expected effects on perceived anger in response to potentially provocative social situations, since self-reported anger decreased in all training conditions. In a previous study of Vassilopoulos et al. (2015), attribution training in a sample of aggressive children not only resulted in a decrease in hostile attributions regarding ambiguous social situations but also led to a decrease in perceived anger in response to hypothetical provocative social situations. Thus, although we found some support for the robustness of the training effects on interpretation bias of intent, we failed to replicate the findings of AlMoghrabi et al. (2018) on aggression-related measures. A possible explanations for this, as mentioned earlier, the changes in interpretation bias were too modest to have an effect on our measures of aggression. Further studies would need to replicate this training procedure, determine its potential and key factors influencing its effectiveness on both bias and aggression reduction prior to drawing any firm conclusions related to the efficacy of our CBM-I training paradigm. However, it was interesting to find that the change in interpretation bias of facial expressions from pre- to post-training did correlate significantly with self-reported physical aggression and anger. Additionally, the change scores of physical aggression correlated significantly with the change scores of interpretation bias of facial expressions in both the positive and negative condition. This result is in line with a previous study that suggested that not only that physical aggression is associated with perceiving anger in facial expressions (Wilkowski & Robinson, 2012), but also that modifying biases in facial affect recognition can be effective in causing changes in self-reported anger and aggression (e.g., Penton-Voak et al., 2013). Wilkowski and Robinson (2012) suggested a possible explanation for the relation between facial perception and physical aggression. They argue that compared to other forms of aggression (e.g., verbal) physical aggression is similar to facial expression perception in the way that they are more present during direct face-to-face communication. This finding builds on similar CBM-I studies targeting facial expressions in aggression (e.g., Hiemstra et al., 2018). It is possible that facial expressions may be a promising factor in increasing the effectiveness of CBM-I training procedures in the context of aggression. By accurately interpreting facial expression of others, it may help aggressive individuals to better interpret others’ intentions and in turn respond more adaptively in social situations.

The current study has a number of limitations. First, all measurements of mood and aggression consisted of self-report, thus it remains unclear whether the effects of training would generalize to aggressive-related responses if participants had to actually participate in a provocative interpersonal situation or engage in a behavioral aggression task (e.g., AlMoghrabi et al., 2018; Hawkins & Cougle, 2013). Second, although the training was successful in causing changes in interpretation bias of intent between the positive and negative group these changes were not generalized to a similar, but distinct, interpretation bias measure (i.e., WSAP-H). Thus, we cannot completely preclude the possibility that the change in interpretation bias of intent is due to some other factors. However, we view the possibility as unlikely since our measures of interpretation bias of intent and facial expressions correlated significantly with the WSAP-H, which provides validity for our interpretation bias measures. Third, the present sample included healthy university students, and therefore, study findings may not be applicable to a clinical sample. Thus, further research is needed to explore the effects of interpretation bias training on reducing aggressive behavior in a clinical sample.

To conclude, the present study is one of the first studies that examined the effect of modifying interpretation bias of intent on attention bias and interpretation bias of facial expressions in the context of aggression. The results have shown that CBM-I can indeed modify interpretation bias of intent in a pro-social direction. However, we found no evidence for the effects of CBM-I training on attention bias, interpretation bias of facial expressions, aggressive behavior and mood. Upon replication and extension of current findings in clinical aggressive samples, this CBM-I training could provide a better understanding of the relation between interpretation bias of intent and aggressive-related cognitive biases such as attention bias. This all might provide more knowledge for the use of CBM-I training as an intervention option in treating aggression.

## Author Note

This article was written within the scope of the dissertation of the first author (AlMoghrabi, 2020).
